# Detection of Indoor High-Density Crowds via Wi-Fi Tracking Data

**DOI:** 10.3390/s20185078

**Published:** 2020-09-07

**Authors:** Peixiao Wang, Fei Gao, Yuhui Zhao, Ming Li, Xinyan Zhu

**Affiliations:** 1 State Key Laboratory of Information Engineering in Surveying, Mapping, and Remote Sensing, Wuhan University, Wuhan 430079, China; peixiao_wang@163.com (P.W.); gaofei_gis@whu.edu.cn (F.G.); 2016206190013@whu.edu.cn (Y.Z.); 2 Institute of Space Science and Technology, Nanchang University, Nanchang 330031, China; liming10307@ncu.edu.cn; 3 Collaborative Innovation Center of Geospatial Technology, Wuhan University, Wuhan 430079, China; 4 Key Laboratory of Aerospace Information Security and Trusted Computing of the Ministry of Education, Wuhan University, Wuhan 430079, China

**Keywords:** high-density crowd location detection, indoor trajectory, Indoor-STAGNES, Indoor-STOPTICS

## Abstract

Accurate detection of locations of indoor high-density crowds is crucial for early warning and emergency rescue during indoor safety accidents. The spatial structure of indoor environments is more complicated than outdoor environments. The locations of indoor high-density crowds are more likely to be the sites of security accidents. Existing detection methods for high-density crowd locations mostly focus on outdoor environments, and relatively few detection methods exist for indoor environments. This study proposes a novel detection framework for high-density indoor crowd locations termed IndoorSRC (Simplification–Reconstruction–Cluster). In this paper, a novel indoor spatiotemporal clustering algorithm called Indoor-STAGNES is proposed to detect the indoor trajectory stay points to simplify indoor movement trajectory. Then, we propose use of a Kalman filter algorithm to reconstruct the indoor trajectory and properly align and resample the data. Finally, an indoor spatiotemporal density clustering algorithm called Indoor-STOPTICS is proposed to detect the locations of high-density crowds in the indoor environment from the reconstructed trajectory. Extensive experiments were conducted using indoor Wi-Fi positioning datasets collected from a shopping mall. The results show that the IndoorSRC framework evidently outperforms the existing baseline method in terms of detection performance.

## 1. Introduction

Indoor environments are the main space for human activities, with research showing that human activities occur indoors approximately 87% of the time [1]. As a result, many indoor spaces host large numbers of people at any point in time. High-density crowds are the primary cause of indoor emergency safety accidents, such as overcrowding and trampling [2,3]. Compared with the outdoor environment, indoor three-dimensional spatial structures are more complicated, and safety accidents are more likely to occur there. Therefore, accurately detecting the locations of these high-density indoor crowds is important for early warning and emergency rescues during instances of indoor safety accidents.

With the rapid development of the Internet, indoor positioning has gradually become a rigid demand [4,5,6]. In recent years, indoor positioning technology has gradually matured and has been applied in our daily life, such as for indoor navigation and indoor location tracking. Concurrently, the available indoor positioning data of indoor users have grown severalfold, becoming a substantial data source for indoor-related research, such as indoor location prediction [7,8,9], indoor association rule mining [10], and indoor positioning methods [11,12,13]. Existing indoor-related research is mainly focused on indoor location services, and there are few studies related to detecting high-density indoor crowd locations. At present, studies related to high-density crowd detection are mainly focused on the outdoor environment. Compared with outdoor trajectories, indoor trajectories are of poor quality and have typical three-dimensional characteristics, which makes it difficult for traditional outdoor high-density crowd detection algorithms to be applied to indoor spaces.

Therefore, a novel high-density location-detection framework for indoor crowds called IndoorSRC (Simplification–Reconstruction–Cluster) is proposed herein. This study uses clustering algorithms to find high-density locations of indoor crowds, thereby providing a scientific basis for indoor emergencies. Based on the characteristics of indoor trajectories, we have made certain improvements to the existing clustering algorithms. The significant contributions of the study are summarized as follows.

(1) A novel indoor spatiotemporal aggregation hierarchical clustering algorithm called Indoor-STAGNES is proposed for detecting the stay points of indoor trajectory and for simplifying indoor movement trajectory.
(2) The Kalman filter algorithm is proposed to reconstruct the indoor trajectory, thereby achieving the required alignment and resampling.
(3) A new indoor spatiotemporal density clustering algorithm called Indoor-STOPTICS is proposed for detecting the location of high-density crowds in the indoor environment from the reconstructed trajectory.
(4) Subsequently, we describe our evaluation of the performance of the IndoorSRC framework using real indoor trajectories. The results demonstrate the advantages of our approach compared to the baseline.

The rest of this paper is organized as follows. In Section 2, a review of the literature focusing on indoor trajectories and detection of high-density locations in outdoor environments is presented. The basic and problem definitions along with a new methodological framework for detecting high-density locations of indoor crowds are described in Section 3. The performances of the frameworks proposed in previous research and this study are compared based on real indoor Wi-Fi positioning data and are presented in Section 4 along with the results. Section 5 provides the conclusion of the study and suggestions for possible further studies are presented.

## 2. Related Work

In this section, we first review the research related to indoor trajectories and, then, review the detection methods for high-density crowd locations in outdoor environments.

Existing research related to indoor trajectories is mainly focused on indoor positioning technology and indoor location services. Indoor positioning technology is used to improve the accuracy of indoor positioning to obtain more accurate indoor movement trajectories. For example, Ye et al. [14] used a hidden Markov model to improve the accuracy of indoor positioning based on traditional fingerprint positioning. Tomazic et al. [15] proposed a confidence interval fuzzy-logic model to improve the accuracy of indoor pedestrian positioning. Indoor location services primarily improve the indoor user experience from multiple perspectives. For instance, Wang et al. [16,17] proposed the Indoor-WhereNext and Markov-LSTM models from the perspectives of group users and individual users based on indoor trajectories of a mall to predict the next location of indoor users and achieved high prediction performance. Li et al. [18] used uncertain historical indoor mobility data to determine the top-k popular indoor semantic locations with the highest flow values. Mou et al. [10] proposed an R-FP-Growth algorithm based on the traditional FP-Growth algorithm to mine association rules among shops in shopping malls, thereby providing indoor location services. Liu el at. [19] designed a graph structure (IT-Graph) that captures indoor temporal variations to return the valid shortest path. Baba el at. [20] proposed the Indoor RFID Multi-variate Hidden Markov Model (IR-MHMM) to capture the uncertainties in indoor RFID data as well as the correlation between moving object locations and object RFID readings. In addition, several scholars have tried to determine high-density crowd locations from indoor trajectories. For example, Li et al. [21] proposed a data-driven approach that finds the top-k indoor density regions using indoor positioning data; however, there are only a few high-density indoor crowd detection methods, making this an ongoing problem in the field.

Detection methods for high-density crowds in outdoor environments are mainly used to find urban hotspots and alleviate traffic congestion. Identifying urban hotspots can reveal the travel characteristics of urban residents. For example, Zheng et al. [22] proposed a grid-based clustering algorithm based on taxi-trajectory data to find popular travel areas and, thus, analyzed the travel patterns of Chongqing residents. Lu et al. [23] presented a visual analysis system to explore the Origin–Destination patterns of hotspots to reveal the potential functions of urban regions. Zhao et al. [24] proposed a trajectory clustering method based on decision graphing and data fields to determine the dynamic pattern of urban hotspots. Easing traffic congestion mainly provides support for urban traffic planning and management. For instance, Li et al. [25] proposed a density-based clustering algorithm called FlowScan to identify high-density traffic locations at road-level to alleviate traffic congestion. Anbaroglu et al. [26] proposed a Non-Recurrent Congestion (NRC) events detection methodology to support the accurate detection of NRC events on large urban road networks. Cheng et al. [27,28] used a data-driven approach to predict changes in traffic flow to alleviate traffic congestion. However, the abovementioned methods mainly focus on high-density crowd detection in outdoor environments. Due to the low quality and three-dimensional characteristics of indoor trajectories, it is difficult to apply these methods directly to indoor environments.

In this study, we propose a novel high-density indoor crowd location detection framework, termed IndoorSRC. Compared with existing methods, the proposed framework is suitable for indoor spaces. It is a lightweight framework that is not only easy to implement but also combines the advantages of multiple clustering algorithms to improve detection performance.

## 3. Materials and Methods

First, it is necessary to define the terms utilized herein and identify the problems to be addressed.

**Definition** **1** **(Indoor** **Trajectory).** *An indoor trajectory,*$traj\;=\;{\left\{pt_{i}\right\}}_{i\;=\;1}^{n}$*, is an ordered sequence of points for*$pt_{i}\;=\;\left(id,t_{i},x_{i},y_{i},f_{i}\right)$*, where*n*is the length of the trajectory,*id*is the length of the trajectory,*$t_{i}$*is a unique user identifier, is the time that*$pt_{i}$*was collected, and*$\left(x_{i},y_{i},f_{i}\right)$*corresponds to the longitude, latitude, and floor, respectively, of the user at time*$t_{i}$.

**Figure 1:**
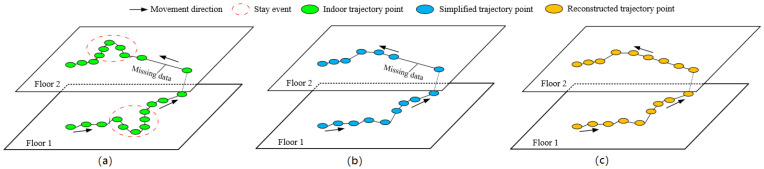
Basic definition: (**a**) raw trajectory of a user; (**b**) simplified trajectory of a user; and (**c**) reconstructed trajectory of a user.

**Definition** **2** **(Simplified** **Trajectory).** *A simplified trajectory,* $\mathrm{sim\_traj}\;=\;{\left\{\mathrm{sim\_p}t_{i}\right\}}_{i\;=\;1}^{k}$ *, simplifies the trajectory points caused by the stay event in the indoor trajectory. As shown in Figure 1b,* $\mathrm{sim\_p}t_{i}\;=\;\left(id,\mathrm{sim\_}t_{i},\mathrm{sim\_}x_{i},\mathrm{sim\_}y_{i},f_{i}\right)$ *is obtained by simplifying the points that are continuous in time and close to each other, where* $\mathrm{sim\_}t_{i}$ *is the average time of the simplified points,* $\left(\mathrm{sim\_}x_{i},\mathrm{sim\_}y_{i}\right)$ *is the center coordinate of the simplified points, and* $f_{i}$ *is the floor on which the user is located.*

**Definition** **3** **(Reconstructed** **Trajectory).** *A reconstructed trajectory,* $\mathrm{rec\_traj}\;=\;{\left\{\mathrm{rec\_p}t_{i}\;\right\}}_{i\;=\;1}^{l}\;=\;{\left\{id,t_{i},{\hat{x}}_{i},{\hat{y}}_{i},f_{i}\right\}}_{i}^{l}$ *, reconstructs the missing data in the simplified trajectory. As shown in Figure 1c,* $t_{i}$ *is the recording time of the reconstructed point* $\mathrm{rec\_p}t_{i}$ *,* $\left(\hat{x},\hat{y}\right)$ *is the coordinate information of the user at time* $t_{i}$ *, and* $f_{i}$ *is the floor on which the user is located.*

**Definition** **4** **(Reconstructed** **Trajectory** **Point** **Set).** *The reconstructed trajectories of all users form a set of reconstructed trajectory points* $DB\;=\;{\left\{\mathrm{rec\_p}t_{i}\right\}}_{i\;=\;1}^{M}$ *, where* M *is the total number of reconstructed trajectory points for all users.*

The research object of this study is the trajectories ${\left\{traj_{i}\right\}}_{i\;=\;1}^{N}$ of the group users in the indoor environment. From the trajectories of the group users, the high-density locations of the crowds in the indoor environment are found, thereby assisting in early warning and emergency rescue during indoor safety accidents. The problem defined in this study is expressed by Equation (1):

(1) $${\left\{l_{i}\right\}}_{i\;=\;1}^{m}\;=\;ℳ\leftarrow {\left\{traj_{i}\right\}}_{i\;=\;1}^{N}$$

where ${\left\{traj_{i}\right\}}_{i\;=\;1}^{N}$ represents the group user trajectory for modeling, N represents the total number of users, ℳ represents the IndoorSRC framework proposed in this study, which is used to detect high-density crowds in the trajectories of group users, and ${\left\{l_{i}\right\}}_{i\;=\;1}^{m}$ represents the high-density locations detected by framework ℳ.

**Figure 2:**
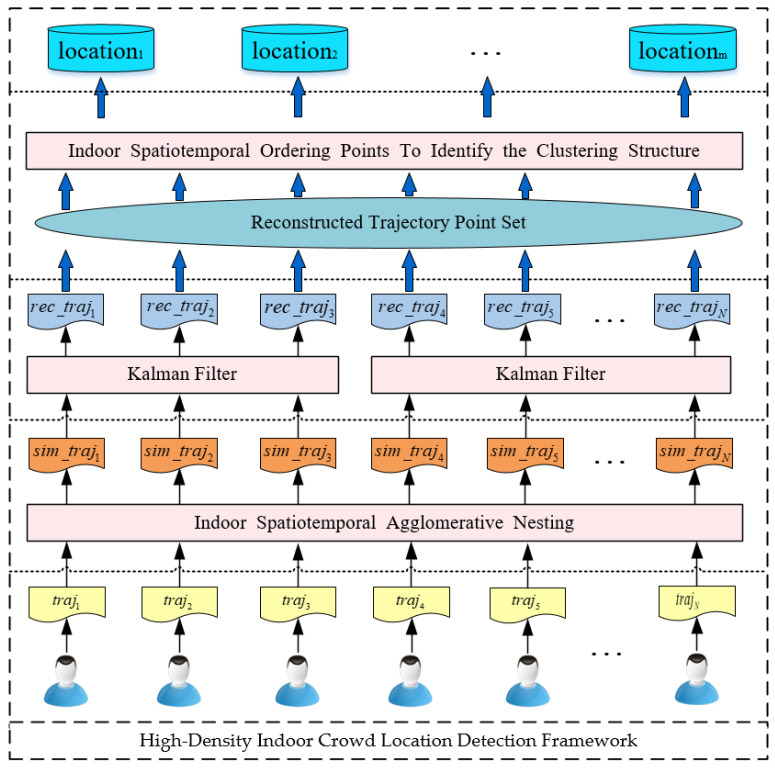
IndoorSRC framework.

The IndoorSRC structure is presented in Figure 2. Based on the bottom-up design principle, our method is divided into three phases: simplification of the indoor movement trajectory; reconstruction of the indoor movement trajectory; and detection of high-density indoor crowd locations. First, a new indoor spatiotemporal agglomeration nesting called Indoor-STAGNES is proposed, which is used to identify the stay point in the indoor trajectory and simplify the indoor movement trajectory. Second, we propose using a Kalman filter algorithm [29,30] to reconstruct the simplified trajectory to align and resample the indoor movement trajectory. Finally, an indoor spatiotemporal density clustering algorithm called Indoor-STOPTICS is proposed to detect the locations of high-density crowds in the indoor environment from the reconstructed trajectory point set.

### 3.1. Simplification of the Indoor Movement Trajectory

The sampling interval of indoor positioning data is heterogeneous; when a user stays in a specific area for a certain period, the mobile terminal will record more trajectory points in the limited area, thereby forming a cluster of trajectory points. If the original trajectory traj is used to directly identify the high-density crowd locations, the high-density *point locations* are often obtained rather than the crowd locations. Therefore, we propose a novel Indoor-STAGNES algorithm to simplify the user trajectory and remove the stay point information from the trajectory.

**Figure 3:**
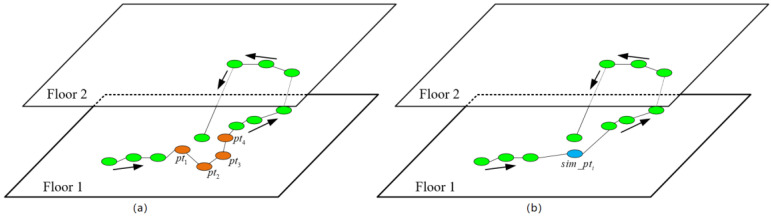
Simplified process of the Indoor-STAGNES algorithm: (**a**) indoor trajectory of a user, (**b**) indoor simplified trajectory of a user.

The Indoor-STAGNES algorithm is an improvement over the traditional agglomerative nesting (AGNES) algorithm. Two major improvements have been made—the addition of time and floor constraints and, consequently, the adjacent spatiotemporal trajectory points (clusters) on the same floor are merged by iteration. Finally, the original $traj\;=\;{\left\{pt_{i}\right\}}_{i\;=\;1}^{n}$ is divided into k disjointed sequential clusters $\left\{C_{1},C_{2},\ldots ,C_{k}\right\}$. The $\mathrm{sim\_p}t_{i}$ is obtained by simplifying the points in cluster $C_{i}$, and k simplified trajectory points are obtained from k clusters, i.e., $\mathrm{sim\_traj}\;=\;{\left\{\mathrm{sim\_p}t_{i}\right\}}_{i\;=\;1}^{k}$. As shown in Figure 3, the cluster $C_{i}\left(pt_{1},pt_{2},pt_{3},pt_{4}\right)$ is iterated into a new cluster and, then, simplified into a trajectory point $\mathrm{sim\_p}t_{i}$. The calculation methods of the time distance and spatial distance between clusters are shown in Equations (2) and (3):

(2) $$spatial_{dist\left(C_{i},C_{j}\right)}\;=\;{\left\|\bar{pt_{i}}-\bar{pt_{j}}\right\|}_{2},\;\;\bar{pt_{i}}\;=\;\frac{1}{\left|C_{i}\right|}\underset{pt_{i}\in C_{i}}{\sum }pt_{i},$$

(3) $$time_{dist\left(C_{i},C_{j}\right)}\;=\;\left|C_{i}.time_{ave}-C_{j}.time_{ave}\right|,\;C_{i}.time_{ave}\;=\;\frac{1}{\left|C_{i}\right|}\underset{pt_{i}\in C_{i}}{\sum }pt_{i}.t,$$

where spatial_dist is used to calculate the spatial distance between $C_{i}$ and $C_{j}$, time_dist is used to calculate the time distance between $C_{i}$ and $C_{j}$, $\bar{pt_{i}}\;$ represents the mean coordinate of cluster $C_{i}$, the number of points in $C_{i}$ is represented by $\left|C_{i}\right|$, and $C_{i}.time_{ave}$ represents the average recording time of the trajectory points in cluster $C_{i}$.

The overall process of Indoor-STAGNES is shown in Algorithm 1.

(1) The indoor trajectory $traj\;=\;{\left\{pt_{i}\right\}}_{i\;=\;1}^{n}$ of the continuous time is input and each trajectory point is initialized as a cluster.
(2) The spatial distance matrix SD and the time distance matrix TD between the clusters are initialized, where $SD_{ij}$ represents the spatial distance between $C_{i}$ and $C_{j}$, and $TD_{ij}$ represents the time distance between $C_{i}$ and $C_{j}$. If $C_{i}$ and $C_{j}$ are not on the same floor, $SD_{ij}$ and $TD_{ij}$ are infinity.
(3) The minimum value $d_{\min}$ in the distance matrix SD under the time threshold $t_{threh}$ is examined. If $d_{\min}$ is smaller than the distance threshold $d_{threh}$, the two nearest clusters are merged, and the spatial distance matrix SD and the time distance matrix TD are updated. Otherwise, step 4 is followed.
(4) The cluster $\left\{C_{1},C_{2},\ldots ,C_{k}\right\}$ is simplified to $\left\{\mathrm{sim\_p}t_{1},\mathrm{sim\_p}t_{2},\ldots ,\mathrm{sim\_p}t_{k}\right\}$ in chronological order.

**Algorithm 1** Indoor Spatiotemporal Agglomerative Nesting**Require:** Individual trajectory: $traj\;=\;{\left\{pt_{i}\right\}}_{i\;=\;1}^{n}$Time threshold: $t_{threh}$Distance threshold: $d_{threh}$**Ensure:** Individual simplified trajectory: $\;\left\{\mathrm{sim\_p}t_{1},\;\mathrm{sim\_p}t_{2},\ldots ,\;\mathrm{sim\_p}t_{k}\right\}$1. Initialize clusters $clsArr\;=\;{\left\{C_{i}\right\}}_{i}^{n}$ based on $traj\;=\;{\left\{pt_{i}\right\}}_{i\;=\;1}^{n}$2. Construct the spatial distance matrix SD and the time distance matrix TD3. Search $d_{min}$ under the time threshold $\;t_{threh}$ in matrix SD4. **while**
$d_{min}\;\leq \;d_{threh}$
**do**5. Search two clusters $cls_{1}$*,*
$cls_{2}$ that need to be merged based on $d_{min}$6. Merge cluster $cls_{1}$ and cluster $cls_{2}$, and update clsArr7. Update matrixes SD and TD based on clsArr8. Search $d_{min}$ under the time threshold $t_{threh}$ in matrix SD9. **for** each cls ∈ clsArr
**do**10. Simplify cluster cls into simplified trajectory point $\mathrm{sim\_p}t_{i}$11. **return**
$\left\{\mathrm{sim\_p}t_{1},\;\mathrm{sim\_p}t_{2},\ldots ,\mathrm{sim\_p}t_{k}\right\}$

### 3.2. Reconstruction of the Indoor Movement Trajectory

The simplified trajectory sim_traj generally reflects the user mobile skeleton; however, it is not suitable for detecting high-density indoor crowd locations. The simplified trajectory sim_traj contains more missing trajectory points. If the simplified trajectory sim_traj is directly used to detect the desired location, the detection performance will be affected to some extent. Therefore, indoor trajectory reconstruction is one of the key steps involved in this process. To complete the missing trajectory points in the simplified trajectory, we proposing using a Kalman filter algorithm to reconstruct the simplified trajectory.

**Figure 4:**
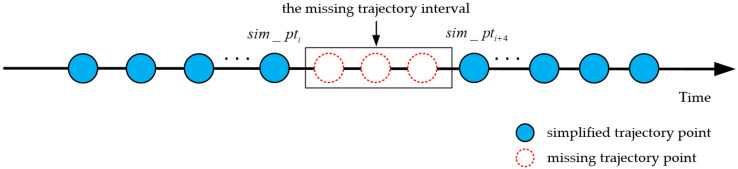
Simplified trajectory with missing trajectory points.

Kalman filtering is a linear optimal estimation algorithm that comprehensively considers measurement data and physical motion models and iteratively estimates the optimal location of a user at each moment, that is, the reconstructed trajectory point rec_pt. The Kalman filtering algorithm reconstructs the indoor movement trajectory in two main stages:

(1) Identification of the missing trajectory points: The number of missing trajectory points in the simplified trajectory are determined according to the sampling interval of the simplified trajectory. As shown in Figure 4, the trajectory interval with a sampling interval that exceeds twice the average sampling interval in the simplified trajectory is regarded as the missing trajectory interval. When the sampling interval of the missing trajectory interval is less than the 95th percentile of the sampling interval, the missing trajectory points need to be reconstructed. The calculation method of the number and time information of the missing trajectory points are shown in Equations (4) and (5):
  (4) $$count\;=\;floor\left(\frac{\mathrm{missing\_interval}}{\mathrm{ave\_interval}}\right)\;+\;1,$$
  (5) $$\mathrm{rec\_p}t_{i\;+\;j}.t\;=\;\mathrm{rec\_p}t_{i}.t\;+\;j\;\;\times \mathrm{ave\_interval},\;\;1\;\leq \;j\;\leq \;count,$$
  where missing_interval represents the sampling interval of the missing trajectory interval, ave_interval represents the average sampling interval of the simplified trajectory points, floor(x) represents the downward rounding function, count represents the number of missing trajectory points, and $\mathrm{rec\_p}t_{i\;+\;j}.t$ represents the time information of the j-th missing trajectory point in the missing trajectory interval (for example, $\mathrm{rec\_p}t_{i\;+\;1}.t$ represents the time information of the first missing trajectory point in the missing trajectory interval).
(2) Reconstruction of the missing trajectory points: The Kalman filtering algorithm iteratively solves the location of the reconstructed trajectory point at each moment, which is mainly divided into two stages: the location prediction and location update stages. In the location prediction stage, the physical motion model is used to predict the location of the next moment according to the optimal location of the previous moment. In the location update stage, the optimal location of the current moment is obtained by correcting the predicted location of the current moment using measurement data and error of the current moment. The iterative process is shown in Equations (6) and (7):
  (6) $$\mathrm{rec\_p}t_{i}.\hat{x}\;=\;kalManFilter\left(\mathrm{rec\_p}t_{i\;-\;1}.\hat{x},\mathrm{sim\_p}t_{i}.x\right),$$
  (7) $$\mathrm{rec\_p}t_{i}.\hat{y}\;=\;kalManFilter\left(\mathrm{rec\_p}t_{i\;-\;1}.\hat{y},\;\mathrm{sim\_p}t_{i}.y\right),$$
  where kalManFilter represents the Kalman filter algorithm, $\left(\mathrm{rec\_p}t_{i}.\hat{x},\mathrm{rec\_p}t_{i}.\hat{y}\right)$ represents the coordinates of the reconstructed trajectory point at the current moment, $\left(\mathrm{rec\_p}t_{i-1}.\hat{x},\mathrm{rec\_p}t_{i\;-\;1}.\hat{y}\right)$ represents the coordinates of the reconstructed trajectory point at the previous moment, and $\left(\mathrm{sim\_p}t_{i}.x,\mathrm{sim\_p}t_{i}.y\right)$ represents the coordinates of the simplified trajectory point at the current moment.

### 3.3. Detection of High-Density Indoor Crowd Locations

The reconstructed trajectory point rec_pt can accurately reflect the movement of a user’s location. By combining all the reconstructed trajectory points of all users, we can analyze the changes in the indoor group user’s location, thereby detecting the locations of high-density indoor crowds. We regard the high-density clusters in the reconstructed trajectory point set DB (Definition 4) as these locations. Therefore, we proposed a novel indoor spatiotemporal ordering point to identify the cluster structure (Indoor-STOPTICS).

**Definition** **5** **(Indoor** **Spatiotemporal** **Neighborhood).** *For* $\mathrm{rec\_p}t_{i}\in DB,$ *the indoor spatiotemporal neighborhood of its indoor space is defined as a cylinder, with* $\epsilon_{1}$ *as* $\epsilon_{2}$ *its radius and as its time window;* $\mathrm{rec\_p}t_{i}$ *is the center of the cylinder,* $N_{\epsilon_{1},\epsilon_{2}}\left(pt_{i}\right)$ *represents a subset of points contained inside the cylinder, and the points in* $N_{\epsilon_{1},\epsilon_{2}}\left(pt_{i}\right)$ *are on the same floor as* $pt_{i}$ *, as defined in Equation (8):*

(8) $$N_{\epsilon_{1},\epsilon_{2}}\left(\mathrm{rec\_p}t_{i}\right)\;=\;\left\{\begin{array}{ccc} \mathrm{rec\_p}t_{j}\in DB & s.t. & \begin{array}{c} sd\left(\mathrm{rec\_p}t_{j},\mathrm{rec\_p}t_{i}\right)\;\leq \epsilon_{1}\\ td\left(\mathrm{rec\_p}t_{j},\mathrm{rec\_p}t_{i}\right)\;\leq \epsilon_{2}\\ \mathrm{rec\_p}t_{j}.f_{j}\;==\;\mathrm{rec\_p}t_{i}.f_{i} \end{array} \end{array}\right\},$$

*where* sd *is used to calculate the spatial distance between* $\mathrm{rec\_p}t_{i}$ *and* $\mathrm{rec\_p}t_{j}$ *,* td *is used to calculate the time distance between* $\mathrm{rec\_p}t_{i}$ *and* $\mathrm{rec\_p}t_{j}$ *, and the number of points in* $N_{\epsilon_{1},\epsilon_{2}}\left(\mathrm{rec\_p}t_{i}\right)$ *is represented by* $\left|N_{\epsilon_{1},\epsilon_{2}}\left(\mathrm{rec\_p}t_{i}\right)\right|$ *.*

**Definition** **6** **(Indoor** **Core** **Trajectory** **Point).** *For* $\mathrm{rec\_p}t_{i}\;\in \;DB$ *, if its indoor spatiotemporal neighborhood* $N_{\epsilon_{1},\epsilon_{2}}\left(\mathrm{rec\_p}t_{i}\right)$ *contains at least* Minpt *indoor trajectory points, that is,* $|N_{\epsilon_{1},\epsilon_{2}}\left(\mathrm{rec\_p}t_{i}\right)|\;>\;Minpt$ *, then,* $\mathrm{rec\_p}t_{i}$ *is called the indoor core trajectory point.*

**Figure 5:**
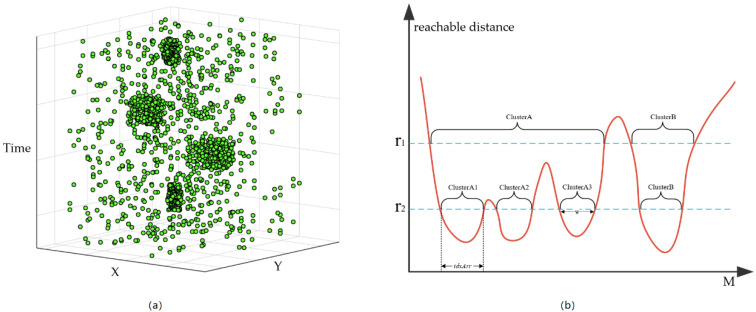
Detection process of the Indoor-STOPTICS algorithm: (**a**) reconstructed trajectory point set DB and (**b**) decision graph of point set DB.

Indoor-STOPTICS is an improved algorithm of spatiotemporal ordering points to identify the clustering structure [31,32]. Indoor-STOPTICS first considers the three-dimensional characteristics of indoor trajectories and adds the floor constraint based on ST-OPTICS (Definition 5). Then, it uses core points (Definition 6) as drivers to determine the set of trajectory points connected with the maximum density of the same floor under spatiotemporal constraints. Unlike traditional density-based spatiotemporal clustering algorithms, the Indoor-STOPTICS algorithm does not explicitly generate clusters, but generates a reachable distance for each data point and an ordered list for analysis to assist in detecting high-density crowd locations in the DB. The calculation method for the reachable distance of each point and the ordered list is the same as in ST-OPTICS [31,32]. As shown in Figure 5, the reconstructed trajectory point set $DB\;=\;{\left\{\mathrm{rec\_p}t_{i}\right\}}_{i\;=\;1}^{M}$ generates an ordered point list $orderList\;=\;{\left\{\mathrm{rec\_p}t_{j}\right\}}_{j\;=\;1}^{M}$ using the Indoor-STOPTICS algorithm. Taking orderList index j as the horizontal axis and $\mathrm{rec\_p}t_{j}$ reachable distance as the vertical axis, a decision graph of DB can be obtained. The auxiliary information of the decision graph can be summarized as follows.

(1) When the spatial radius of the Indoor-STOPTICS algorithm is $\epsilon_{1}\;=\;r_{1}$, two clusters, ClusterA and ClusterB, can be detected from the set DB.
(2) When the spatial radius of the Indoor-STOPTICS algorithm is $\epsilon_{1}\;=\;r_{2}$, ClusterA is split into three small clusters, namely ClusterA1, ClusterA2, and ClusterA3. Thus, a total of four clusters can be identified from the set DB.
(3) When the spatial radius of the Indoor-STOPTICS algorithm is $\epsilon_{1}\;=\;r_{2}$, the trajectory points included in each cluster can be obtained by the corresponding horizontal axis index sequence. For example, the horizontal axis index sequence corresponding to ClusterA1 is idxArr and the trajectory points included in ClusterA1 can be expressed as ${\left\{orderList\left[i\right]\right\}}_{i\in idxArr}$.
(4) When the spatial radius of the Indoor-STOPTICS algorithm is $\epsilon_{1}\;=\;r_{2}$, the cluster density can be approximated by the width of the cluster. For example, the density of ClusterB can be represented by w. When w is wider, the density of the cluster is greater.

## 4. Results

### 4.1. Data Preparation

#### 4.1.1. Data Sources

The experimental data mainly included Wi-Fi positioning data from a shopping mall in Jinan City, China. The indoor Wi-Fi positioning data covered eight floors of the shopping mall from 23 December, 2017, to 30 December 2017. Approximately 2 million indoor movement trajectories and 30 million indoor trajectory points were collected every day. The positioning accuracy was approximately 3 m, and trajectory points with a sampling interval of 1–5 s accounted for more than 70% of the collected data points. Table 1 lists the unique identifier of the user, record upload time, the user’s (X, Y) coordinates, and the unique floor identifier.

#### 4.1.2. Data Preprocessing

The original Wi-Fi data were collected via fingerprint positioning technology. First, multiple Wi-Fi access points (APs) were deployed in the study area and, then, the coordinate information of each AP was calculated iteratively. After the determination of coordinate information of each AP, the research area was divided into multiple grids that do not overlap; then, fingerprint information from each grid was obtained to construct a fingerprint database. When a mobile terminal enters the coverage area of APs, the mobile terminal matches the signal strength of received AP with the fingerprint database to determine the specific location of the terminal. Because of the unstable signal of the mobile terminal and the artificial shutdown of the Wi-Fi signal, it was easy to generate abnormal, erroneous, and invalid data. There were three types of noise in our dataset:

(1) The coordinate abnormal point. If the trajectory point fell outside the study area, it was regarded as a coordinate abnormal trajectory point.
(2) The trajectory point generated by fixed devices. If a user trajectory remained in the same area for more than eight hours, it was regarded as a trajectory point generated by fixed devices.
(3) The floor abnormal point. If a trajectory point of the user jumped between different floors within a short period, it was regarded as a floor abnormal point.

### 4.2. Evaluation Metrics

In this study, we regarded the clusters in the reconstructed trajectory point set DB as the high-density indoor crowd locations and used crowd density (CD), point density (PD), and running time as the quantitative evaluation indexes of the IndoorSRC framework. The CD and PD can be defined by Equations (9) and (10), respectively:

(9) $$\mathrm{CD}\;=\;\overset{m}{\underset{i}{\sum }}\frac{{\mathrm{CrowdNum}}_{i}}{V_{i}}\;\times \;\frac{\Delta t}{m},$$

(10) $$\mathrm{PD}\;=\;\overset{m}{\underset{i}{\sum }}\frac{{\mathrm{PointNum}}_{i}}{V_{i}}\;\times \;\frac{\Delta t}{m},$$

where m represents the number of clusters detected by the IndoorSRC framework, $V_{i}$ represents the volume of a certain cluster (i.e., the convex hull volume of the three-dimensional point set), ${\mathrm{CrowdNum}}_{i}$ represents the number of users in a cluster, ${\mathrm{PointNum}}_{i}$ represents the number of trajectory points in a cluster, and Δt represents the time step.

### 4.3. Variable Estimation

The hyperparameters of the IndoorSRC framework primarily included parameters in the Indoor-STAGNES and Indoor-STOPTICS algorithms. The Indoor-STAGNES algorithm has two auxiliary functions. First, it simplifies the user trajectory and reduces the number of trajectory points, thereby reducing the running time of the Indoor-STOPTICS algorithm. Second, it ensures that a single user contains only one trajectory point in a particular spatiotemporal neighborhood. Thus, the detection performance of the IndoorSRC framework predominantly depends on the Indoor-STOPTICS algorithm. Hence, we set the distance threshold $d_{threh}$ and the time threshold $t_{threh}$ to fixed values in the Indoor-STAGNES algorithm, wherein the distance threshold $d_{threh}$ was fixed to 5 m with reference to the average distance between indoor shops and the time threshold $t_{threh}$ was fixed to 4 min.

**Figure 6:**
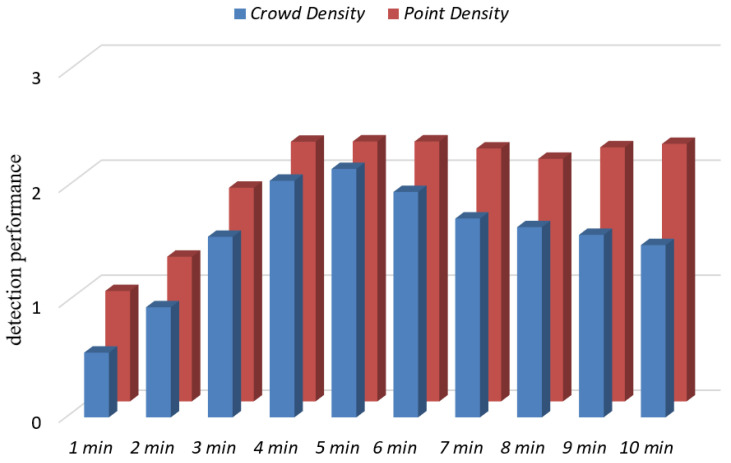
Impact of parameters ($\epsilon_{2}$) on IndoorSRC.

The hyperparameters of Indoor-STOPTICS mainly include the radius $\epsilon_{1}$, time window $\epsilon_{2}$, and minimum number of points MinPts. In the Indoor-STDBSCAN algorithm, the main test time window $\epsilon_{2}$ influences the detection performance. To determine the parameters in Indoor-STOPTICS, the control variable method was used to obtain the combination of parameter values and the best detection performance. In the parameter estimation phase, the radius $\epsilon_{1}$ was set to infinity for generating the decision graph, time window $\epsilon_{2}$ was the best parameter found in [1 min,2 min,3 min,…,10 min], and minimum number of MinPts was set to 5 × ln(M) [33], where M represents the number of points in the set DB. Figure 6 shows the effect of the time window $\epsilon_{2}$ on detection performance. The crowd density first increased and then decreased, whereas the point density first increased and then stabilized. This is due to the fact that when the time window $\epsilon_{2}$ is greater than the time threshold $t_{threh}$ in the Indoor-STAGNES algorithm, the probability of including multiple points of a user in the neighborhood (Definition 5) is higher in the Indoor-STOPTICS algorithm. This, therefore, leads to a decrease in the crowd density; the two auxiliary functions of the Indoor-AGNES algorithm is also confirmed, to a certain extent. In this study, we eventually fix the time window $\epsilon_{2}$ to 5 min.

### 4.4. IndoorSRC Framework Performance

**Figure 7:**
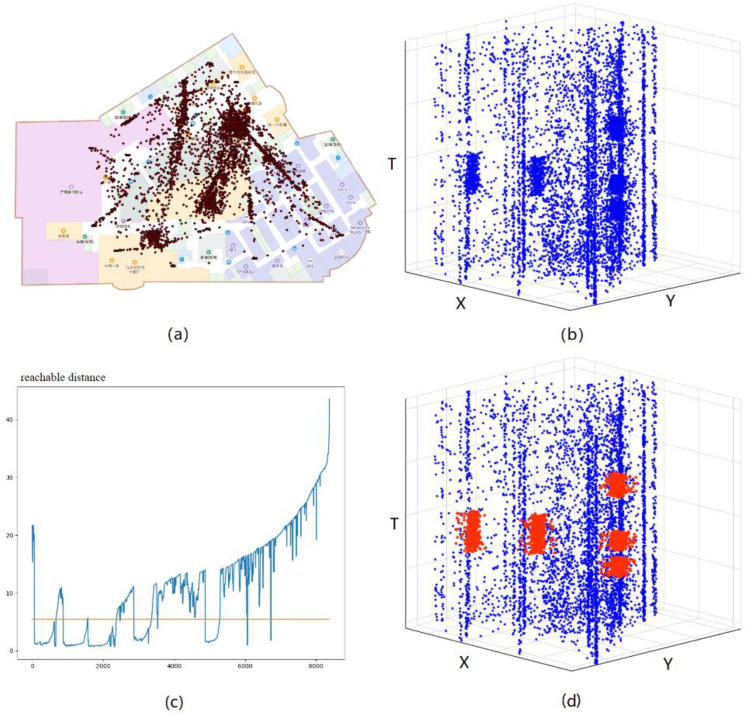
Detection results of the IndoorSRC framework: (**a**) floor graph of set DB; (**b**) spatiotemporal prism graph of set DB; (**c**) decision graph of set DB; and (**d**) detection results of set DB.

The IndoorSRC detection results on specific floors obtained after determining the optimal combination of parameters, using 11:00–16:00 as the research time, are presented graphically in Figure 7. The reconstructed trajectory point set DB shows an obvious aggregation pattern in different regions and at different times. After drawing the decision graph of the set DB, the spatial coordinates and time of the high-density crowd location can be detected. For example, when the distance threshold $\epsilon_{1}$ = 6 m, there will be eight clusters in the set DB; upon further determining the width of each cluster, five high-density crowd locations will be obtained. Table 2 shows the spatial and temporal information of each high-density crowd location. These locations are mostly crowded at noon and are primarily located in the dining area. For example, “Food Shangjia” is part of the food court inside the shopping mall and three short-period–high-density crowd locations were formed there at noon. “Fisherman’s lamp” and “Chinese Restaurant” are restaurants inside the shopping mall; long-period–high-density crowd locations were formed here.

### 4.5. Comparison with Baselines

To verify the performance of the proposed IndoorSRC framework, it was compared with the existing ST-OPTICS, and the experimental results were analyzed from the perspectives of CD, PD, and running time.

**Figure 8:**
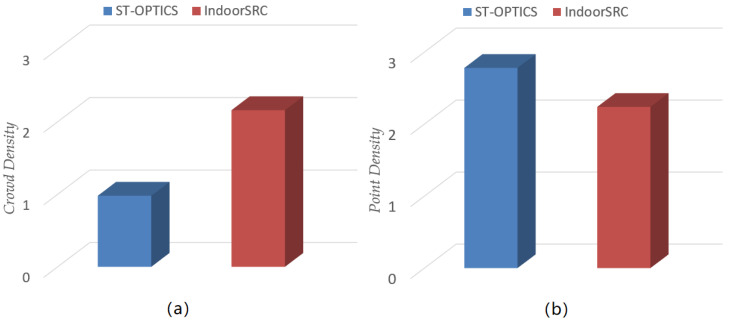
Comparison of the point density and crowd density with the baseline method: (**a**) crowd density comparison, (**b**) point density comparison.

Figure 8 shows the comparison results of the point and crowd densities with the baseline. From these perspectives, the crowd density detected by the IndoorSRC algorithm is much higher than that calculated by ST-OPTICS. This is because there is more stay-point information in the indoor movement trajectory. If ST-OPTICS is used to identify high-density crowd locations directly, the clusters obtained are mostly “high-density point locations” rather than “high-density crowd locations.” The density of points detected by the IndoorSRC algorithm is slightly lower than that by ST-OPTICS as the IndoorSRC framework simplifies and reconstructs the indoor movement trajectory so that the adjacent trajectory points in the reconstructed trajectory are farther apart. This makes the trajectory points more sparse, resulting in a slight decrease in the point density. Therefore, the IndoorSRC framework is more suitable for detecting high-density indoor crowd locations.

**Figure 9:**
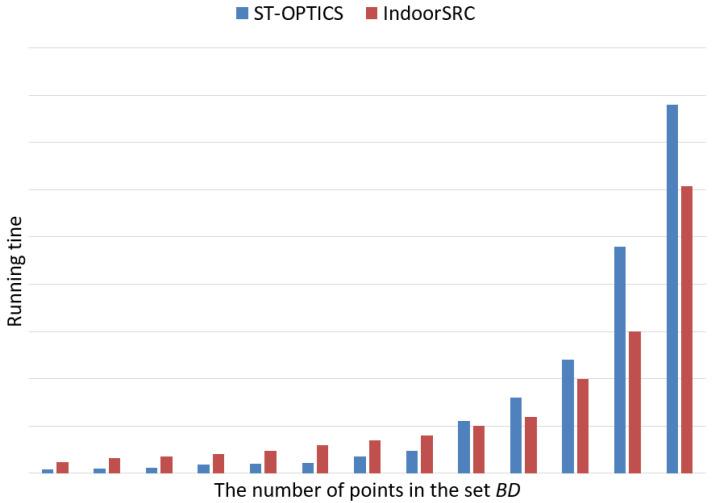
Comparison of the running time with the baseline approach.

Figure 9 shows the comparative results of the running time with the baseline approach. In this study, we compared the running time of the entire framework with baseline and not the running time of a simple single component, such as Indoor-STOPTICS or Indoor-STAGNES. From the perspective of the framework running time, when the number of users is small, the running time of the IndoorSRC framework is slightly higher than that of the ST-OPTICS algorithm, as the former simplifies and reconstructs the indoor movement trajectory, which increases the running time of the framework. As the number of users increases, the running time of the ST-OPTICS algorithm exceeds that of the IndoorSRC framework. This is because, when the number of trajectory points is very large, although the IndoorSRC framework simplifies and reconstructs the indoor movement trajectory, it greatly reduces the number of trajectory points, and the time consumed by simplification and reconstruction is far less than that consumed by direct detection.

## 5. Conclusions and Future Work

Accurate and robust detection of high-density indoor crowd locations is of great significance for early warning and emergency rescue during indoor safety accidents. Compared with the outdoor environment, the spatial structure of the indoor environment is more complicated, and tightly packed indoor crowds are more likely to cause security accidents. In this paper, the IndoorSRC framework is proposed to detect high-density indoor crowd locations. First, Indoor-STAGNES is proposed to detect the stay points of the indoor trajectory and simplify it. Then, the use of a Kalman filter algorithm to reconstruct the indoor trajectory is proposed. Finally, Indoor-STOPTICS is proposed to detect the location of high-density crowds in the indoor environment.

Experimentally, a two-week real indoor trajectory was used to verify the detection performance of the proposed framework. First, we used the control variable method to obtain the optimal parameter combination of the IndoorSRC framework. Afterward, we analyzed the predictive performance of the IndoorSRC framework using the dataset. Then, we conducted a comparison with the existing ST-OPTICS algorithm. Compared with the existing approach, the IndoorSRC framework considerably improved the detection performance in running time and crowd density, which demonstrates the efficiency of the IndoorSRC framework.

The following problems need to be investigated in the future. This study considered the high density of indoor crowds as the only necessary condition for indoor congestion, trampling, and other safety accidents; however, additionally, the direction of user movement can affect the occurrence of indoor safety accidents to a great extent. For example, when the crowd density is high and the users’ walking directions are the same, accidents often do not occur. When the crowd density is high and the users’ walking directions are disordered, there is more probability of an accident occurring. Therefore, future studies should introduce additional constraints, such as direction, to further improve the practicality of the IndoorSRC framework.

## Figures and Tables

**Table 1:** Sample table of user trajectory data.

User ID	Date and Time	X (m)	Y (m)	Floor ID
2813BF ***	2017–12–29 09:25:58	130,219 ***	43,904 ***	2
2813BF ***	2017–12–29 09:26:01	130,219 ***	43,903 ***	2
2813BF ***	2017–12–29 09:26:05	130,219 ***	43,904 ***	2
……	……	……	……	……
2813BF ***	2017–12–29 20:18:48	130,219 ***	43,904 ***	5
2813BF ***	2017–12–29 20:18:51	130,219 ***	43,904 ***	5

*** means data omitted.

**Table 2:** Indoor high-density crowd locations and time characteristics.

	High-Density Crowd Locations	Time
Location 1	Food Shangjia	11:31–11:56
Location 2	Food Shangjia	12:07–12:34
Location 3	Chinese Restaurant	12:16–13:32
Location 4	Fisherman’s lamp	12:20–13:24
Location 5	Food Shangjia	14:14–14:17
